# Heart rate acceleration at relative workloads during treadmill and overground running for tracking exercise performance during functional overreaching

**DOI:** 10.1038/s41598-020-71597-4

**Published:** 2020-09-03

**Authors:** Clint R. Bellenger, Rebecca L. Thomson, Eileen Y. Robertson, Kade Davison, Maximillian J. Nelson, Laura Karavirta, Jonathan D. Buckley

**Affiliations:** 1 Alliance for Research in Exercise, Nutrition and Activity (ARENA), Sansom Institute for Health Research, University of South Australia, Adelaide, SA 5001 Australia; 2 South Australian Sports Institute, Adelaide, Australia; 3 Polar Electro Oy, Kempele, Finland; 4 Gerontology Research Center and Faculty of Sport and Health Sciences, University of Jyväsky-lä, Jyväskylä, Finland

**Keywords:** Cardiovascular biology, Circulation

## Abstract

Maximal rate of heart rate (HR) increase (rHRI) as a measure of HR acceleration during the transition from rest to exercise, or during an increase in workload, tracks exercise performance. rHRI assessed at relative rather than absolute workloads may track performance better, and a field test would increase applicability. This study therefore aimed to evaluate the sensitivity of rHRI assessed at individualised relative workloads during treadmill and overground running for tracking exercise performance. Treadmill running performance (5 km time trial; 5TTT) and rHRI were assessed in 11 male runners following 1 week of light training (LT), 2 weeks of heavy training (HT) and a 10-day taper (T). rHRI was the first derivative maximum of a sigmoidal curve fit to HR data collected during 5 min of treadmill running at 65% peak HR (rHRI65%), and subsequent transition to 85% peak HR (rHRI85%). Participants ran at the same speeds overground, paced by a foot-mounted accelerometer. Time to complete 5TTT likely increased following HT (ES = 0.14 ± 0.03), and almost certainly decreased following T (ES = − 0.30 ± 0.07). Treadmill and field rHRI65% likely increased after HT in comparison to LT (ES ≤ 0.48 ± 0.32), and was unchanged at T. Treadmill and field rHRI85% was unchanged at HT in comparison to LT, and likely decreased at T in comparison to LT (ES ≤ − 0.55 ± 0.50). 5TTT was not correlated with treadmill or field rHRI65% or rHRI85%. rHRI65% was highly correlated between treadmill and field tests across LT, HT and T (r ≥ 0.63), but correlations for rHRI85% were trivial to moderate (r ≤ 0.42). rHRI assessed at relative exercise intensities does not track performance. rHRI assessed during the transition from rest to running overground and on a treadmill at the same running speed were highly correlated, suggesting that rHRI can be validly assessed under field conditions at 65% of peak HR.

## Introduction

Knowledge of training status in athletes is important in identifying the accumulation of training-induced fatigue (i.e. overreaching), and facilitating subsequent adjustments in training load to ensure such fatigue is transient and ultimately results in supercompensatory performance improvements (i.e. functional overreaching [FOR])^1,2^. Recently, a novel marker of autonomic heart rate (HR) regulation which reflects HR acceleration during the transition from rest to exercise or during an increase in workload, termed the maximal rate of HR increase (rHRI), has been investigated as a marker of training status, and shown to be sensitive for detecting both acute fatigue^3^ and a state of FOR^4–8^ in trained runners and cyclists.

Most recently however, Bellenger et al.^7^ showed that an individual’s level of physical conditioning may affect the sensitivity of rHRI for tracking training-induced changes in exercise performance. Specifically, in an attempt to investigate the effect of varying absolute exercise intensities on rHRI assessment and its ability to track changes in exercise performance in response to changes in training load, Bellenger et al.^7^ assessed rHRI during the transition from rest to a light intensity running speed (8 km.h^−1^), and during the subsequent transition from 8 km.h^−1^ to a heavier intensity running speed (13 km.h^−1^). rHRI tracked changes in exercise performance at both 8 km.h^−1^ (r = − 0.84) and during the transition to 13 km.h^−1^ (r = − 0.52), but only in a sub-group of athletes who were less well conditioned (i.e. required a longer time to complete a five km time-trial). In this sub-group, the running speeds elicited ~ 10% higher steady-state HR compared with the better conditioned athletes at both 8 km.h^−1^ (~ 65 vs ~ 55% of peak HR) and 13 km.h^−1^ (~ 85 vs ~ 75% of peak HR). These findings suggested that rHRI assessed at individualised relative workloads of 65% and 85% of peak HR, rather than set absolute workloads, might elicit an optimal physiological stress that may enable rHRI to more sensitively track changes in exercise performance in all athletes. In previous studies^4–7,9^, rHRI was assessed at absolute workloads to maximise its practical application, since it would be simpler to have all athletes exercise at the same workloads rather than at individualised workloads. However, some practicality may need to be traded-off to maximise sensitivity.

In addition, research to date has assessed rHRI using bicycle or treadmill ergometers to better standardise exercise intensity^4–7,9^. Given that many athletes do not have access to ergometers, and/or perform the majority of their training in the field, the development of a field test for the assessment of rHRI would enhance the practical application of this parameter for monitoring training status across a broad range of athletes.

Consequently, the primary aim of this study was to evaluate whether rHRI assessed at individualised relative workloads can track changes in performance resulting from transient fatigue and subsequent supercompensation. In addition, this study aimed to validate a field test for the assessment of rHRI. Given the aforementioned research by Bellenger et al.^7^, it was hypothesised that rHRI assessed at individualised relative workloads would track changes in performance more sensitively than rHRI assessed at absolute workloads demonstrated in previous rHRI research^4–6^, and that rHRI assessed during overground running would track performance as sensitively as treadmill running.

## Methods

### Participants

Fifteen male runners were recruited from running and triathlon clubs in Adelaide, South Australia. Participants were eligible for inclusion if they displayed no known signs or symptoms of cardiometabolic disease, were currently completing 40 km or more of running per week, self-reported as injury free in the three months prior to undertaking the study, and could complete a five kilometre treadmill time trial (5TTT) in less than 23 min. Ethical approval was approved by the University of South Australia’s Human Research Ethics Committee in accordance with the Declaration of Helsinki, and volunteers provided written informed consent prior to participating.

### Experimental overview

Two pre-study familiarisation sessions allowed participants to be habituated with the testing procedures, and determine their peak HR during a 5TTT. Given that rHRI was to be determined at relative running speeds based upon percentages of peak HR, familiarisation sessions also allowed for evaluation of these speeds. Specifically, participants ran at three different speeds based on perception of their current physical fitness for 4 min each. Steady-state HR (mean HR over the final 60 s) for each workload was plotted against the corresponding running speed, and the equation for the regression line representing this relationship was determined. Using the peak HR obtained during 5TTT, the absolute HRs (bpm) at 65% and 85% of peak HR and the corresponding running speeds required to elicit these HRs were calculated. This process was repeated during both familiarisation sessions and the data were averaged. The running speeds were fixed for each testing visit thereafter, such that running speeds were constant within individuals, but differed between individuals, in order to elicit a similar relative physiological stress across all participants. rHRI (assessed while running at these speeds on a treadmill and during overground running) and 5TTT performance were measured after one week of light training (LT—baseline), two weeks of heavy training (HT—overreached state) and 10 days of taper (T—recovered and adapted state). Assessments occurred at the same time of day on the day after completion of the final training session for each training phase (i.e. 24 h) and the order of assessments of overground or treadmill running was randomised. Participants were instructed not to complete any exercise on the days they were to be tested, and were encouraged to maintain their daily routine (including diet and hydration) throughout the study. Environmental conditions (ambient temperature, airflow and precipitation) were controlled in the laboratory setting, however ambient temperature was not able to be controlled during field test rHRI assessment.

### Assessments

**Figure 1:**
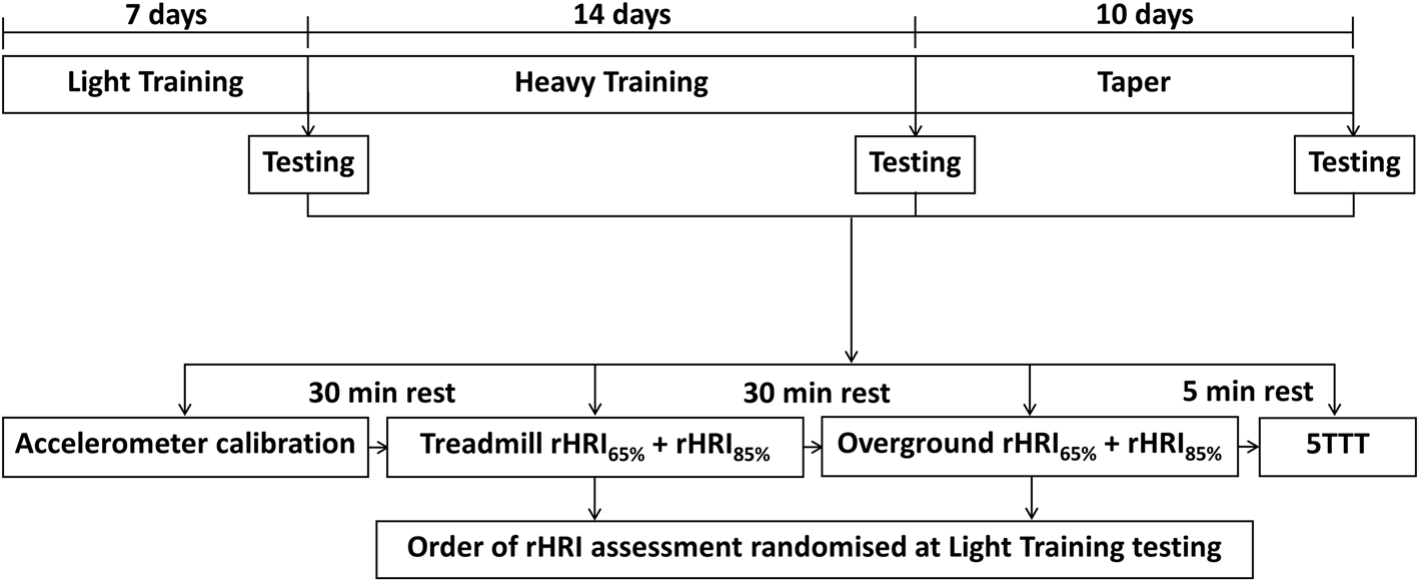
Training intervention and testing protocol flowchart. *min* minute, *rHRI*_*65%*_ maximal rate of heart rate increase assessed at a running speed eliciting ~ 65% of peak HR (5 min in duration); *rHRI*_85%_, maximal rate of heart rate increase assessed at a running speed eliciting ~ 85% of peak HR (5 min in duration), *5TTT* 5-km treadmill time-trial.

During experimental testing visits, participants completed three submaximal running tasks; one for the calibration of a foot-worn accelerometer (Polar s3 + stride sensor, Polar Electro Oy, Kempele, Finland) used to pace overground running, and two for the assessment of rHRI at 65% and the transition to 85% of peak HR when running both on a treadmill and overground (Fig. 1).

Calibration of the foot-worn accelerometer required participants to run continuously for 6 min on a calibrated treadmill (Trackmaster TMX425CP, Full Vision Inc., Newton, KS, USA) at the two speeds determined during familiarisation to elicit 65% and 85% of peak HR (3 min at each speed). The accelerometer provided instantaneous running speed feedback via wireless transmission to a HR monitor (RS800CX, Polar Electro Oy, Kempele, Finland). The distance covered in each 3 min period was recorded from the treadmill’s control panel, and compared to the distance covered in each period according to the accelerometer. A correction factor (treadmill distance divided by accelerometer distance) was then applied to the accelerometer via software available in the HR monitor in order to calibrate it against the treadmill.

Following calibration of the accelerometer, participants ran continuously for a total of 10 min to determine rHRI at each of the two speeds designed to elicit 65% (rHRI_65%_) and the transition to 85% (rHRI_85%_) of peak HR (5 min at each speed) on a treadmill and in the field (overground running). The order in which treadmill and field assessments occurred was randomised at baseline, and this order was held constant at each visit thereafter.

During treadmill-derived rHRI, participants were instructed without warning to begin exercise to avoid an anticipatory rise in HR^10^. At exercise onset, they lowered themselves onto the already moving treadmill belt and ran at the initial speed for 5 min, before the treadmill speed was increased for the next 5 min.

Field-derived rHRI was obtained while running continuously for 10 min (5 min at each speed) around an indoor stadium which had a wooden parquetry floor. Plastic cones denoted an approximate 85 m oval-shaped running track with running speed governed by the accelerometer, which was paired with a wrist-worn HR monitor and programmed to sound an audible beep if actual running speed varied from the target speed by more than ± 0.5 km.h^−1^. The field test was programmed as an exercise mode within the HR monitor, and audible beeps also indicated when to start the exercise task, when to transition from the initial speed to the second speed and when to cease exercise.

After 5 min of rest, rHRI assessments were followed by a 5TTT where the time taken to run five kilometres on a motorised treadmill was recorded as the measure of exercise performance. Participants chose their preferred starting speed during familiarisation which remained constant across visits. Participants were blinded to running time and speed, but could see the distance covered and were free to adjust the treadmill speed as desired to complete five km in the fastest time possible. Reliability of 5TTT in a separate group of trained runners was determined to be excellent (CV = 1.3%^11^).

To assist in confirming a state of functional overreaching, participants completed a Daily Analysis of Life Demands for Athletes (DALDA) questionnaire throughout the training intervention. The DALDA is a subjective measure of training tolerance scored on a three point scale (worse than normal, normal, better than normal), and is sensitive to perturbations in various parameters (e.g. diet, social/work life, sleep, fatigue, muscle soreness, etc.) resulting from periods of overload training in athletes^12,13^.

### rHRI calculation

RR intervals were recorded for maximal HR curve resolution during rHRI testing. A 5-component sigmoidal curve was fit (Eq. 1) to the HR data recorded during the 30 s preceding exercise onset (or preceding the change in speed when determining rHRI_85%_), and throughout the subsequent 5 min of steady-state exercise.

1 $$\hat{y} = a \, + \frac{b}{{1 + f_{x} \cdot e^{{c\left( {d - x^{\prime}} \right)}} + \left( {1 - f_{x} } \right) \cdot e^{{e\left( {d - x^{\prime}} \right)}} }}$$

where

$$f_{x} = \frac{1}{{1 + e^{{ - \overline{C}_{f} \left( {d - x^{\prime}} \right)}} }}$$

defines a logistic weighting function varying smoothly between 0 and 1, centered about *d* so long as *c* and *e* are of the same sign, and where the mean curvature of *f* is given by

$$\overline{C} = \frac{2 \cdot c \cdot e}{{c + e}}$$

rHRI (bpm^.^s-1) was the first derivative maximum of this curve (Eq. 2) obtained using the Solver function in Excel (Microsoft Corporation, NY, USA).

2 $${\varvec{x}} = \frac{{{\varvec{b}} \times \left( {{\varvec{c}} + {\varvec{e}}} \right)}}{8}$$

where ***a*** = lower HR plateau, ***b*** = range of HR response, ***c*** = upper curvature parameter, ***d*** = time at which half of the range of HR response was attained, ***e*** = lower curvature parameter.

Pre-exercise HR (mean HR during the 30 s prior to exercise), steady-state HR (mean HR during the final 60 s of exercise) and change in HR (steady-state HR minus pre-exercise HR) were also calculated during rHRI assessment.

### Training intervention

The training intervention used in this study has been described elsewhere^12^ but briefly, LT was designed to allow participants to be rested and recovered from any pre-study training prior to undergoing HT, which was designed to induce fatigue from which participants would not recover prior to testing on the day following the final training session (i.e. a state of overreaching). The subsequent T was designed to allow recovery and adaptation to training to occur. HR data were recorded at 15 s intervals during training for determination of training load using TRIMP (arbitrary units [AU])^14^ (duration in minutes multiplied by % of peak HR), and training intensities were based on percentages of peak HR determined during familiarisation.

### Statistical analysis

Data were analysed using PASW Statistics 18.0 (SPSS, Chicago, IL, USA) and presented as mean ± SD, and ES with 90% confidence intervals. Data were log transformed before analysis to reduce bias from non-uniformity of error^15^. Outcome measures were compared using repeated measures analysis of variance with Bonferroni post-hoc comparison and statistical significance set at P < 0.05. Data were also analysed using magnitude-based inferences^15^, with changes in variables after each training period analysed using a modified statistical spreadsheet^16^, which calculated ES between time-points of interest using pooled standard deviation^17^. Threshold values for ES statistics were ≤ 0.2 (trivial), > 0.2 (small), > 0.6 (moderate), > 1.2 (large), > 2.0 (very large), and > 4.0 (extremely large)^15^. Probabilities to establish whether the true differences were lower, similar, or higher than the smallest worthwhile change were also calculated. Chances of higher or lower differences were evaluated qualitatively as: < 1%, almost certainly not; 1–5%, very unlikely; 5–25%, unlikely; 25–75%, possibly; 75–95%, likely; 95–99%, very likely; and > 99%, almost certain. If the chance of higher and lower differences was > 5%, the true difference was assessed as unclear. Within-subject correlations between rHRI and performance across testing time-points were evaluated using univariate analysis of covariance^18^, with r values evaluated as: 0.0–0.1, trivial; 0.1–0.3, small; 0.3–0.5, moderate; 0.5–0.7, large; 0.7–0.9, very large; 0.9–1.0, nearly perfect. Multiple linear regression was utilised to evaluate the effect of changes in HR variables (i.e. the mathematically modelled *a*, *b*, *c*, *d* and *e* constants, pre-exercise HR, steady-state HR, change in HR and peak HR) on changes in rHRI. Absolute agreement between treadmill and field-derived measures was determined through limits of agreement analysis^19^, while relative agreement was determined using the intra-class correlation (ICC), with ICCs evaluated using the aforementioned classifications.

## Results

### Participants

Fourteen of the 15 recruited participants completed the study (age 35.8 ± 10.0 years; height 1.78 ± 0.09 m; body mass 77.3 ± 10.0 kg); one participant was unable to tolerate the demands of HT and withdrew from the study. Three of the 14 completed participants were considered to be acutely fatigued, but not overreached, as they did not experience a decline in 5TTT performance after HT^20^, and were excluded from further analysis so as not to attenuate the true effect of HT on variables of interest in those participants experiencing FOR, as recommended by Bellenger et al.^21^. A sub-group analysis was not performed on these three participants given the small sample size. Thus, data for 11 participants were included for analysis (age 37.5 ± 8.2 years; body mass 78.5 ± 10.3 kg; self-reported weekly running distance 46.2 ± 16.8 km in the previous six months).

### Effect of training on running performance

**Figure 2:**
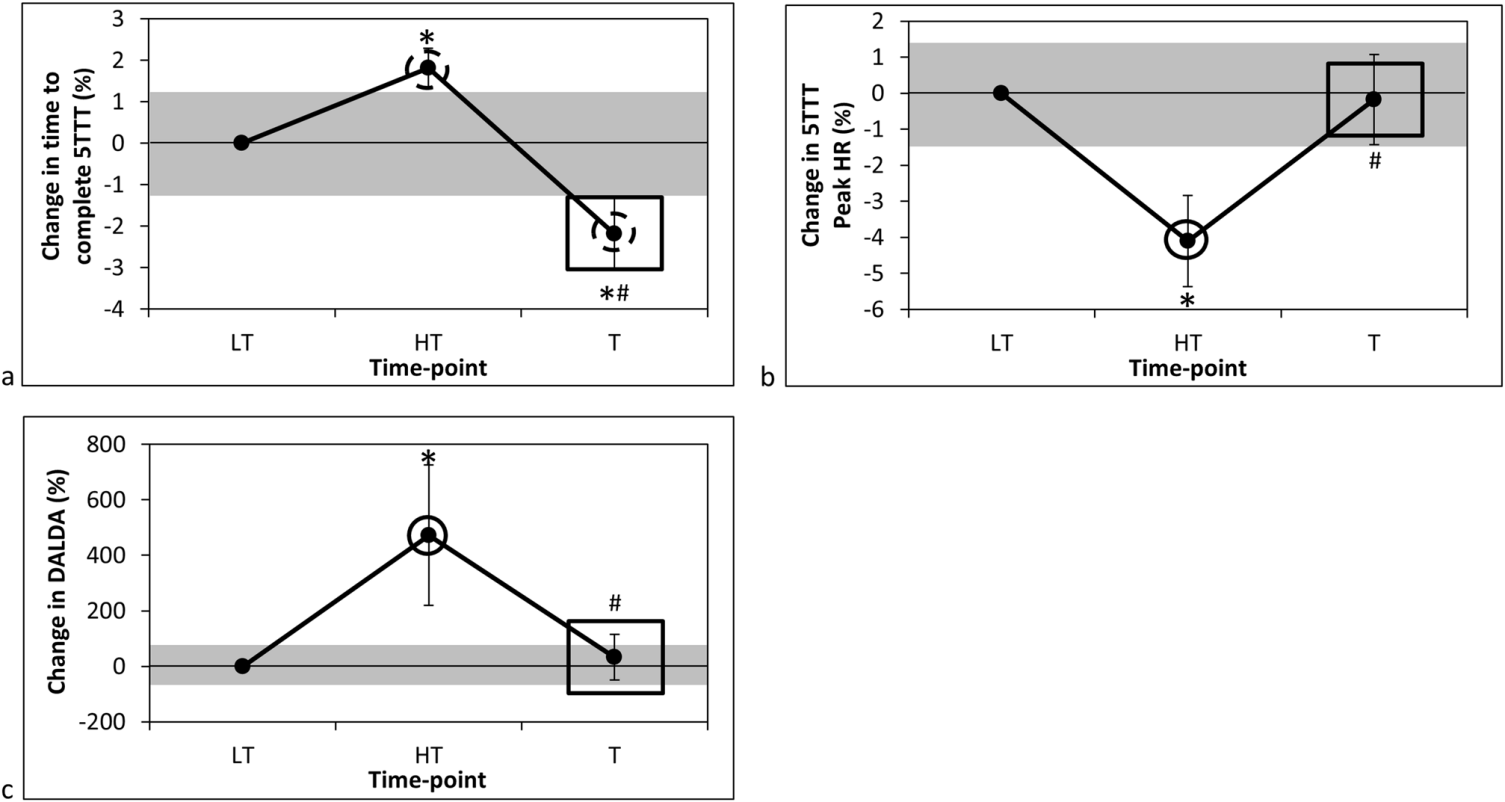
Percentage change in (**a**) time to complete 5TTT, (**b**) peak HR during 5TTT and (**c**) DALDA ‘worse than normal’ score from LT. Data are mean ± 90% confidence level. *DALDA* daily analysis of life demands for athletes questionnaire, *HR* heart rate, *HT* heavy training, *LT* light training, *T* tapering, *5TTT* 5-km treadmill time-trial. Grey shaded areas represent the smallest worthwhile change. Dashed circle, very likely chance of practically meaningful difference in value from LT; continuous circle, almost certain chance of practically meaningful difference in value from LT; continuous rectangle, almost certain chance of practically meaningful difference in value from HT; *significantly different (P < 0.05) from LT; #significantly different (P < 0.05) from HT.

Daily TRIMP almost certainly increased from 2,740 ± 301 AU at LT to 5,182 ± 890 AU at HT (ES ± 90% confidence interval = 3.85 ± 0.77; P < 0.001) and then almost certainly decreased to 2,028 ± 407 AU from HT to T (ES = − 5.78 ± 0.71; P < 0.001). The time taken to complete 5TTT was 19:35 ± 2:21 min:s at LT, which very likely increased to 19:56 ± 2:21 min:s at HT (ES = 0.14 ± 0.03; P < 0.001), and then almost certainly decreased to 19:09 ± 2:13 min:s from HT to T (ES = − 0.30 ± 0.07; P < 0.001). Overall, these changes resulted in a very likely improvement in running performance from LT to T (− 00:26 ± 0:20 min:s; ES = − 0.17 ± 0.07; P = 0.005; Fig. 2a). Peak HR during 5TTT was 184 ± 11 bpm at LT, and almost certainly decreased to 176 ± 9 bpm at HT (ES = − 0.75 ± 0.24; P = 0.001), before almost certainly increasing to 184 ± 8 bpm at T (in comparison to HT; ES = 0.72 ± 0.22; P < 0.001; Fig. 2b).

### Effect of training on subjective training tolerance

The number of ‘worse than normal’ scores on the DALDA was 1.3 ± 1.0 units at LT. This value almost certainly increased to 7.3 ± 3.4 units at HT (ES = 2.54 ± 0.62; P = 0.001), before almost certainly decreasing to 2.2 ± 2.1 units at T (ES = − 2.16 ± 0.64; p = 0.004; Fig. 2c).

### Effect of training on HR parameters

HR parameters are shown in Table 1. Notably, treadmill and field-derived rHRI_65%_ very likely increased after HT in comparison to LT (ES = 0.33 ± 0.21; P = 0.18 and ES = 0.48 ± 0.32; P = 0.17, respectively). Pre-exercise HR during all assessments of rHRI likely to almost certainly decreased at HT in comparison to LT (ES ≤ − 0.62 ± 0.29; P ≤ 0.05), and then likely to very likely increased at T in comparison to HT during treadmill-derived rHRI_65%_ (ES = 0.79 ± 0.47; P = 0.03) and rHRI_85%_ (ES = 0.40 ± 0.40; P = 0.33), and field-derived rHRI_65%_ (ES = 0.86 ± 0.60; P = 0.08). Steady-state HR during all assessments of rHRI very likely to almost certainly decreased after HT in comparison to LT (ES ≤ − 0.70 ± 0.29; P ≤ 0.008). At T, steady-state HR during rHRI_85%_ assessment likely to very likely remained decreased in comparison LT (ES ≤ − 0.50 ± 0.41; P ≤ 0.15). Differences between treadmill and field-derived rHRI_65%_ and rHRI_85%_ (and their associated pre-exercise and steady-state HRs) in response to training were unclear (ES ≤ 0.42 ± 0.51; P ≥ 0.08).

**Table 1:** Effect of training on heart rate parameters.

Exercise mode	HR parameter	LT	HT	T
Treadmill	rHRI_65%_ (bpm^.^s-1)	5.22 ± 3.34	6.41 ± 4.37^a^	5.83 ± 3.83
	rHRI_65%_ pre-exercise HR (% peak HR)	39.7 ± 3.8	36.4 ± 3.5^a^*	39.6 ± 3.9^b^^#^
	rHRI_65%_ steady-state HR (% peak HR)	66.7 ± 3.2	64.2 ± 3.2^a^*	65.2 ± 3.4^a^
	rHRI_85%_ (bpm^.^s-1)	0.91 ± 0.27	0.85 ± 0.25	0.77 ± 0.26^a^
	rHRI_85%_ pre-exercise HR (% peak HR)	66.8 ± 3.0	64.5 ± 3.7^a^*	66.0 ± 3.5^b^
	rHRI_85%_ steady-state HR (% peak HR)	84.5 ± 1.9	81.2 ± 2.2^a^*	82.3 ± 2.3^a,b^
Field	rHRI_65%_ (bpm^.^s-1)	4.93 ± 3.14	6.29 ± 3.00^a^	5.44 ± 2.99
	rHRI_65%_ pre-exercise HR (% peak HR)	40.6 ± 3.4	35.9 ± 3.8^a^*	38.9 ± 2.6^a,b^
	rHRI_65%_ steady-state HR (% peak HR)	64.7 ± 2.3	61.8 ± 1.7^a^*	63.3 ± 2.9^a^
	rHRI_85%_ (bpm^.^s-1)	1.62 ± 1.31	1.20 ± 0.58	1.06 ± 0.39^a^
	rHRI_85%_ pre-exercise HR (% peak HR)	65.2 ± 2.1	62.4 ± 2.2^a^*	63.4 ± 2.9^a^
	rHRI_85%_ steady-state HR (% peak HR)	85.8 ± 4.6	81.9 ± 4.7^a^*	83.2 ± 4.1^a^

*bpm*^*.*^*s-1* beats per minute per second, *HR* heart rate, *HT* heavy training, *LT* light training, *rHRI*_*65%*_ maximal rate of heart rate increase assessed at a running speed eliciting ~ 65% of peak HR, *rHRI*_*85%*_ maximal rate of heart rate increase assessed at a running speed eliciting ~ 85% of peak HR, *T* tapering.

*Significantly different (P < 0.05) from LT.

^#^Significantly different (P < 0.05) from HT.

^a^Practically meaningful difference from LT.

^b^Practically meaningful difference from HT.

### Agreement between treadmill and field-derived HR parameters

Agreement between treadmill and field-derived HR parameters are shown in Table 2. Treadmill-derived rHRI_85%_ was likely lower than its field-derived measure at LT, HT and T (ES ≤ − 0.67 ± 0.53; P ≤ 0.14). Limits of agreement analysis indicated that the precision of the difference between treadmill and field-derived measures of rHRI_65%_ and rHRI_85%_ (minimum of ± 67.9%) was greater than the coefficient of variation for rHRI assessment in general (i.e. 6.0%^5^), such that a practically meaningful difference between these measures may be evident. The ICC as a measure of relative agreement between treadmill and field-derived measures was large to very large across LT, HT and T (r ≥ 0.63) for rHRI_65%_, and trivial to moderate (r ≤ 0.42) for rHRI_85%_.

**Table 2:** Agreement between treadmill and field-derived HR parameters.

HR parameter	Variable	LT	HT	T
rHRI_65%_ (bpm^.^s-1)	ICC	0.69	0.63	0.82
	Bias (field–treadmill) absolute	− 0.29	− 0.12	− 0.39
	Bias (field–treadmill) %	− 5.50	− 15.73	− 2.04
	LOA absolute	± 5.82	± 7.26	± 5.50
	LOA %	± 67.87	± 112.39	± 89.14
rHRI_85%_ (bpm^.^s-1)	ICC	− 0.05	0.42	− 0.19
	Bias (field–treadmill) absolute	0.66^a^	0.33^a^	0.30^a^
	Bias (field–treadmill) %	93.55*	44.64*	58.90*
	LOA absolute	± 2.65	± 1.02	± 0.95
	LOA %	± 392.22	± 135.98	± 171.48
rHRI_65%_ pre-exercise HR (% peak HR)	ICC	0.76	0.89	0.84
	Bias (Field–Treadmill) Absolute	0.91	− 0.45	− 0.78
	Bias (Field–Treadmill) %	2.63	− 1.19	− 1.58
	LOA Absolute	± 5.08	± 3.66	± 4.10
	LOA %	± 13.70	± 10.19	± 10.67
rHRI_85%_ pre-exercise HR (% peak HR)	ICC	0.68	0.74	0.67
	Bias (field–treadmill) absolute	− 1.57^a^^#^	− 2.13^a^^#^	− 2.56^a^^#^
	Bias (field–treadmill) %	− 2.25^a^^#^	− 3.15^a^^#^	− 3.76^a^^#^
	LOA absolute	± 4.48	± 4.85	± 5.57
	LOA %	± 6.26	± 6.97	± 8.08
rHRI_65%_ steady-state HR (% peak HR)	ICC	0.62	0.28	0.59
	Bias (field–treadmill) absolute	− 1.92^a^^#^	− 2.40^a^^#^	− 1.90^a^
	Bias (field–treadmill) %	− 2.76^a^^#^	− 3.56^a^^#^	− 2.79^a^
	LOA absolute	± 5.20	± 6.13	± 5.91
	LOA %	± 7.34	± 9.38	± 8.77
rHRI_85%_ steady-state HR (% peak HR)	ICC	0.18	0.38	0.58
	Bias (field–treadmill) absolute	1.28	0.71	0.90
	Bias (field–treadmill) %	1.54	0.87	1.08
	LOA absolute	± 8.94	± 8.18	± 6.31
	LOA %	± 10.71	± 10.41	± 7.77

Analysis performed on raw data.

*bpm*^*.*^*s-1* beats per minute per second, *HR* heart rate, *HT* heavy training, *ICC* intra class correlation, *LOA* limits of agreement, *LT* light training, *T* tapering, *rHRI*_*65%*_ maximal rate of heart rate increase assessed at a running speed eliciting ~ 65% of peak HR, *rHRI*_*85%*_ maximal rate of heart rate increase assessed at a running speed eliciting ~ 85% of peak HR.

^#^Significant difference (P < 0.05).

^a^Likely chance of practically meaningful difference in value between treadmill and field assessments.

Treadmill-derived steady-state HR (during rHRI_65%_) and pre-exercise HR (during rHRI_85%_) were likely to very likely higher than their field-derived measures at LT, HT and T (ES ≥ 0.52 ± 0.41; P ≤ 0.06). Limits of agreement for the difference between treadmill and field-derived pre-exercise and steady-state HR during rHRI_65%_ and rHRI_85%_ was greater than the coefficient of variation for these parameters (i.e. 3.3% for pre-exercise HR and 1.4% for steady-state HR [in the present cohort]), such that a practically meaningful difference between treadmill and field-derived measures may be evident. ICCs ranged from small to very large (r = 0.18–0.89).

### Correlations between variables of interest

Multiple linear regression showed that 94 ± 16% (P < 0.001) of the variance in treadmill-derived rHRI_65%_ change between LT and HT was explained by a model including changes in the parameters utilised to calculate rHRI (i.e. the *e*, *c* and *b* constants from a 5 component sigmoidal curve model; beta ± 90% confidence interval = 6.92 ± 1.61; P < 0.001, 7.25 ± 2.60; P = 0.001 and 0.13 ± 0.06; P = 0.005, respectively). However, 81 ± 23% (P = 0.001) of the variance in rHRI_65%_ change between LT and HT was explained by a model including changes in the *e* and *c* constants only (7.33 ± 2.28; P = 0.001 and 4.67 ± 3.24; P = 0.045, respectively), and 68 ± 26% (P = 0.002) of the same variance was explained by a model including changes in the *e* constant only (7.42 ± 2.80). Similarly, changes in the *e* (4.48 ± 0.69; P < 0.001), *c* (3.25 ± 0.87; P < 0.001) and *b* (0.03 ± 0.01; P = 0.001) constants accounted for 98 ± 10% (P < 0.001) of the variance in treadmill-derived rHRI_85%_ change between LT and HT, while changes in the *e* (3.92 ± 1.11; P < 0.001) and *c* (3.98 ± 1.39; P = 0.002) constants alone accounted for 92 ± 18% (P < 0.001) of the variance, and changes in the *e* constant alone accounted for 71 ± 24% (5.27 ± 1.84; P = 0.001) of the variance in this parameter.

Within-subject analysis (using LT, HT and T) revealed that 5TTT was not correlated with either rHRI_65%_ or rHRI_85%_ when derived from treadmill or field exercise tasks (r ≤ 0.19; P ≥ 0.37).

## Discussion

This study aimed to determine whether rHRI assessed at relative workloads could track changes in exercise performance resulting from overload training and subsequent taper. Additionally, a field-based protocol for the assessment of rHRI was evaluated by extending the treadmill methodology to a novel overground running protocol. In contrast to previous research which had assessed rHRI at absolute workloads, rHRI assessed at relative workloads of 65% of peak HR and the transition from 65 to 85% peak HR did not track training-induced changes in performance when assessed during either treadmill or overground running. However, there was large to very large correlation (relative agreement) between treadmill and field-derived measures of rHRI_65%_, but poor relative and absolute agreement between treadmill and field-derived measures of rHRI_85%_, suggesting that rHRI can be assessed in the field when transitioning from rest to a moderate exercise intensity.

Based on recent findings of Bellenger et al.^7^, where rHRI assessed at the same absolute workload tracked exercise performance more strongly in less fit athletes, in whom the same absolute load represented a higher relative load, it was hypothesised that assessing rHRI at relative rather than absolute exercise intensities would improve the sensitivity of rHRI for tracking changes in exercise performance. However, rHRI assessed at 65% peak HR and the transition from 65 to 85% peak HR did not track these changes as anticipated. Of particular interest, was the finding of increased rHRI_65%_ following HT, which is in direct contrast to previous research showing a slowing in rHRI in response to training-induced fatigue^4–8^. Given that the initial and rapid increase in HR during the transition from rest to exercise primarily results from withdrawal of parasympathetic modulation^22^, it may be hypothesised that the apparent increase in resting parasympathetic modulation following HT (as evidenced by the reduced pre-exercise HR) contributed to the unexpected increase in rHRI_65%_. This appears to be supported by the earliest research on rHRI, where small to moderate reductions in rHRI (ES − 0.33 to − 0.65) following a period of HT occurred in the absence of any change in pre-exercise HR (ES − 0.05 to 0.14)^4,5^. In other studies, rHRI has been slowed (ES − 0.40^7^) or remained unchanged (ES 0.06^6^) following HT, despite small reductions in pre-exercise HR (ES − 0.40 to − 0.43). Interestingly however, small to moderate increases in rHRI (ES 0.33 and 0.48 in the present study, and ES 0.64 in Bellenger et al.^7^) occurred concurrently with moderate to large reductions in pre-exercise HR (ES − 0.82 to − 1.32). Together, these data suggest that overreaching interventions inducing larger reductions in pre-exercise HR result in faster rHRI. Unfortunately, it is difficult to explain why the discrepancy in these magnitudes of change in pre-exercise HR has occurred, since the training interventions utilised in these studies to induce a state of overreaching have been similar. While the magnitude of performance decrement in response to these interventions has been somewhat varied (ES − 0.14 to − 0.75), there is no pattern to suggest that studies inducing a greater amount of fatigue (i.e. a greater performance decrement) resulted in a greater reduction in pre-exercise HR.

The present study also demonstrated (through multiple linear regression) that 94–98% of the change in rHRI following HT may be attributed to the concurrent changes in the *b* (range of HR response), *c* (upper curvature parameter) and *e* (lower curvature parameter) constants in a 5-compontent sigmoidal curve model, which is intuitive considering these variables are utilised to calculate rHRI. Of particular interest however, was that changes in the *c* and *e* constants combined, or the *e* constant alone, were the primary determinants of rHRI (accounting for 67–92% of the change in rHRI). Mathematically, the *c* and *e* constants represent curvature parameters of the modelled HR response at the onset of exercise. Theoretically, the *c* curvature parameter represents the initial and rapid acceleration in HR at the onset of exercise, perhaps indicative of the rapid withdrawal of parasympathetic modulation that is known to occur at exercise onset^22^, while the *e* curvature parameter represents the subsequent slower acceleration in HR as exercise intensity increases, which may be more reflective of increased sympathetic modulation^22^. The physiological meaning of changes in these curvature parameters following training however, is presently unknown, but elucidation of their meaning may provide a foundation for greater understanding of how rHRI is modulated in response to exercise training.

The current study also investigated the potential for rHRI assessed through a field test to track changes in exercise performance. While precision of bias (i.e. limit of agreement analysis) suggested that the absolute differences between values of treadmill and field-derived rHRI_65%_ may be large enough to be considered practically meaningful, the large to very large ICCs, indicating good relative agreement between methods, suggested that the training response was similar between the two assessment conditions. With regard to rHRI_85%_, precision of bias again suggested practically meaningful differences between measures, however trivial to moderate ICCs also suggest that the between-participant ordering of values was not consistent for each assessment condition. Together, these results suggest that the present study’s field test assessment of rHRI_65%_ is valid, but the assessment of rHRI_85%_ is not.

It is not immediately clear why field-derived rHRI_65%_ was more valid than field-derived rHRI_85%_, however may be explained by limitations of this study. Specifically, the accelerometer utilised has the potential to provide running speed feedback in real-time, but only after it has been accurately calibrated. Unfortunately, only a single calibration factor may be entered to the accelerometer, however two running speeds were required to be calibrated in the present study, and due to the difference in stride characteristics at these different speeds, two different calibration factors were also required. Thus, in order to run the two-stage test continuously, the average of the two calibration factors was applied, meaning that both speeds may have been slightly different to those ran during treadmill assessment of rHRI. Consequently, since the present study’s field test assessment of rHRI_65%_ demonstrated acceptable relative agreement, but rHRI_85%_ did not, it may be postulated that field-derived rHRI_85%_ was more impacted upon by the average calibration factor than field-derived rHRI_65%_. Future research may investigate alternative means for field-based assessments of rHRI.

Additionally, it should be acknowledged that while the indoor stadium provided controlled conditions with regard to airflow (speed and direction) and precipitation, the ambient temperature was not controlled by thermostat. Thus, while the relatively short duration of the study (~ 5 weeks) allowed each participant to be tested in a similar season of weather, the presence of extreme changes in weather conditions may have affected physiological measures during the assessment of field-derived rHRI.

## Conclusion

In contrast to previous rHRI research which has shown that rHRI assessed at absolute exercise intensities tracks exercise performance, the present study failed to identify within-subject correlations between exercise performance and rHRI assessed at relative exercise intensities. This suggests that assessment of rHRI using relative exercise intensities may not be appropriate for tracking exercise performance. Future research may investigate the physiological meaning of the curvature parameters calculated in rHRI quantification, since this would provide a foundation for greater understanding of how rHRI is modulated in response to exercise training. Additionally, the present study found that rHRI assessed when transitioning from rest to running overground and on a treadmill at the same running speed were highly correlated, suggesting that rHRI can be validly assessed under field conditions.

## Data Availability

The datasets generated during and/or analysed during the current study are available from the corresponding author on reasonable request.
